# Changes in the blood cyclosporine level after switching from voriconazole to isavuconazole in a patient with aplastic anemia: insights from physiologically based pharmacokinetic model simulation and the Adverse Event Reporting System database study

**DOI:** 10.3389/fmicb.2025.1525991

**Published:** 2025-02-24

**Authors:** Chihiro Shiraishi, Hideo Kato, Kazuko Ino, Takuya Iwamoto

**Affiliations:** ^1^Department of Pharmacy, Mie University Hospital, Mie, Japan; ^2^Division of Clinical Medical Science, Department of Clinical Pharmaceutics, Mie University Graduate School of Medicine, Mie, Japan; ^3^Department of Hematology and Oncology, Faculty of Medicine, Mie University Hospital, Mie, Japan

**Keywords:** cyclosporine, isavuconazole, drug–drug interaction, physiologically based pharmacokinetic model simulation, the Adverse Event Reporting System database study

## Abstract

**Introduction:**

Isavuconazole, a broad-spectrum triazole approved by the United States Food and Drug Administration (FDA) in 2015, moderately inhibits cytochrome P450 3A4. Although antifungal agents are often used concomitantly with cyclosporine, the effect of switching from voriconazole to isavuconazole on the blood cyclosporine level remains unclear.

**Case:**

A 63-year-old Japanese male was administered oral cyclosporine (10:00 and 21:00) for severe aplastic anemia. Following pneumonia with positive *Aspergillus* antigen and an elevated β-D-glucan level, antifungal therapy was initiated. After switching from voriconazole (10:00 and 21:00) to isavuconazole (approximately 08:00), the blood cyclosporine level decreased by more than half. Although the blood cyclosporine level decreased after switching to isavuconazole, the dose of cyclosporine was not increased because of its possible effect on renal function. Considering the inhibitory effects on the gastrointestinal tract, a physiologically based pharmacokinetic analysis estimated that isavuconazole increased the area under the curve (AUC) and *C*_max_ of cyclosporine by 1.48-fold and 1.84-fold, respectively, although assuming no change in gastrointestinal metabolism, these effects were minimal. For interaction with voriconazole considering gastrointestinal metabolism, the predicted increases in AUC and *C*_max_ were 3.74-fold and 3.86-fold, respectively. The FDA Adverse Event Reporting System database included 9,144 reports on cyclosporine and 174 on cyclosporine with voriconazole, but none concomitant with isavuconazole. The reporting odds ratios for cyclosporine and isavuconazole could not be assessed because of insufficient reports.

**Conclusion:**

The interaction of isavuconazole with cyclosporine was weaker than that with voriconazole. Maintaining a two-hour dosing interval between isavuconazole and cyclosporine may minimize gastrointestinal drug interactions.

## 1 Introduction

Currently, antifungal agents available for the treatment of invasive fungal infections include polyenes, azoles, echinocandins, and pyrimidine analogs. Second-generation triazoles such as voriconazole have an extended antifungal spectrum. However, their use is often limited by variable bioavailability, severe adverse events, significant drug–drug interactions, and the emergence of resistance (Kato et al., 2016). Therefore, effective and well-tolerated treatment is required for clinical practice.

Isavuconazole, a new extended-spectrum triazole approved by the United States Food and Drug Administration (FDA) in 2015, is used to treat invasive aspergillosis (Maertens et al., 2016). Due to its excellent bioavailability and broad-spectrum activity, isavuconazole is an effective antifungal treatment (Cornely et al., 2019). The SECURE trial demonstrated that isavuconazole is not inferior to voriconazole as the primary treatment for invasive pulmonary aspergillosis, is better tolerated than voriconazole, and has fewer drug-related adverse events (Maertens et al., 2016). The 9th European Conference on Infections in Leukemia, the European Society for Clinical Microbiology and Infectious Diseases, and the European Confederation of Medical Mycology recommend isavuconazole along with voriconazole as the first-line treatment for invasive aspergillosis (Tissot et al., 2017; Ullmann et al., 2018).

Isavuconazole is a moderate inhibitor of cytochrome P450 3A4 (CYP3A4) with fewer drug–drug interactions than voriconazole, a strong CYP3A4 inhibitor (Miceli and Kauffman, 2015). Previous reports indicated that isavuconazole weakly inhibits midazolam and testosterone, which are CYP3A4 substrates (Townsend et al., 2017). A Phase I study demonstrated that concomitant use of isavuconazole increased the area under the curve (AUC) and *C*_max_ of cyclosporine 300 mg (NEORAL^®^ oral capsules, Novartis Pharmaceuticals Corp., East Hanover, New Jersey) by 1.3-fold and 1.1-fold, respectively (Groll et al., 2017). This information is included in the European Medicines Agency prescribing information for isavuconazole (European Medicines Agency, 2024), but has not been described in the FDA prescribing information (Astellas Pharma US Inc., 2024). Consequently, there are no definitive recommendations for cyclosporine in patients receiving isavuconazole. In addition, there have been no reports of cyclosporin-associated adverse events due to interaction with isavuconazole. Furthermore, despite the requirement for a transition between azole antifungals due to toxicity in clinical settings, there is only one report on changes in the blood cyclosporine level when transitioning from voriconazole to isavuconazole (Kozuch et al., 2024). The previous report showed that the blood cyclosporine level/dose-normalized body weight (C/D) ratio in two patients who were switched from voriconazole to isavuconazole, decreased to 52% of baseline on Day 3, after switching, and then remained at 55% ± 11% of the baseline (Kozuch et al., 2024). However, detailed information on the route of administration, drug dosing interval, and adverse events were not provided.

We illustrate one case showing the effect of switching the concomitantly administered triazole antifungal agent from voriconazole to isavuconazole on the blood cyclosporine level to recommend an appropriate dose adjustment of cyclosporine. In addition, a physiologically based pharmacokinetic (PBPK) model simulation was conducted to assess the potential interactions between isavuconazole and cyclosporine and between voriconazole and cyclosporine. Furthermore, we investigated the adverse events associated with cyclosporine when combined with voriconazole or isavuconazole using the FDA Adverse Event Spontaneous Reporting System (FAERS) database. The FAERS database collects and publishes voluntary reports of adverse events reported worldwide. With more than 14 million reports, it is the largest available database of its kind.

## 2 Case

A 63-year-old Japanese male (body weight: 45 kg) with a history of smoking (Brinkman index of 630), hypertension, and severe aplastic anemia, received 60 mg/day of oral cyclosporine [cyclosporine capsules (SANDOZ), Sandoz K.K., Japan] at 10:00 and 21:00 on Day X. The prescription also included prednisolone, which interacts with CYP3A4. He did not consume grapefruit or St. John’s wort for several days as they interact with cyclosporine. There were no problems with medication adherence. Anti-human rabbit thymocyte immunoglobulin was administered from Day X + 1 to Day X + 5.

**Figure 1:**
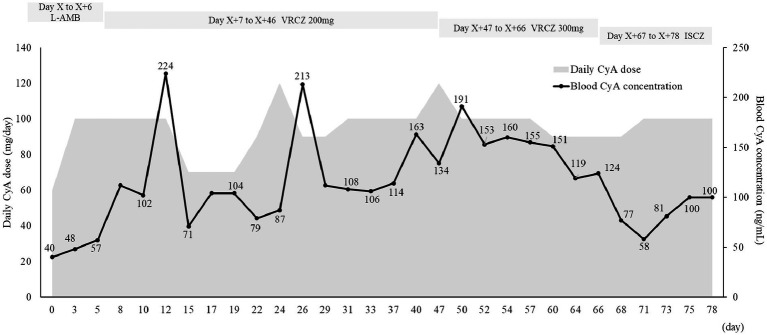
Time-related change in the blood cyclosporine level. CyA, cyclosporine; ISCZ, isavuconazole; L-AMB, liposomal amphotericin B; VRCZ, voriconazole.

The patient developed pneumonia, tested positive for *Aspergillus* antigen (2.7), and had an elevated β-D-glucan level (59.0 pg/mL) on Day X-59. Chest computed tomography was performed, revealing findings suggestive of pneumonia. The patient was admitted to another hospital with pancytopenia and shock, along with elevated alanine transaminase (ALT) levels [Common Terminology Criteria for Adverse Events (CTCAE) Grade 4]. Considering the liver function, liposomal amphotericin B (4 mg/kg/day) was initiated. After liver function improved, the treatment was switched to voriconazole. However, an increase in ALT levels (CTCAE Grade 1) was observed, leading to a subsequent switch back to amphotericin B. Liposomal amphotericin B was continued until Day X + 6. During this period, the blood cyclosporine level ranged from 40 to 57 ng/mL and the C/D ratio ranged from 26 to 36 (ng/mL)/(mg/kg) (Figure 1 and Supplementary Table 1). The C/D ratio was calculated as follows: C/D (ng/mL)/(mg/kg) = (blood cyclosporine level)/(daily cyclosporine dose per body weight). Due to worsening *Aspergillus* infection symptoms and the development of leukopenia (CTCAE Grade 4; 150/μL), thrombocytopenia (CTCAE Grade 3; 27,000/μL), and anemia (CTCAE Grade 3; 7.5 g/dL), liposomal amphotericin B was replaced with voriconazole on Day X + 7. Voriconazole (200 mg/day) was administered orally at 10:00 and 21:00. Considering liver function, voriconazole was initially administered at a reduced dose of 200 mg/day. After the initiation of voriconazole, the blood cyclosporine level increased to 224 ng/mL and the C/D ratio reached 99 (ng/mL)/(mg/kg) on Day X + 12. No other factors were identified that increased blood cyclosporine levels. *Aspergillus* antigen was measured regularly, and it remained negative continuously after Day X + 15. On Day X + 40, the voriconazole trough concentration was 0.6 μg/mL. Since there were no changes in liver function, the voriconazole dose was increased to 300 mg/day, the recommended dose according to the package insert. The blood voriconazole level was not measured after the increase in the dose. Although the *Aspergillus* antigen became negative, the possibility of new nodular shadows was noted. Considering the upward trend in β-D-glucan levels, voriconazole was switched to isavuconazole on Day X + 66. Isavuconazole (CRESEMBA^®^ for infusion, Basilea Pharmaceutica International Ltd., Allschwil) was administered intravenously at 200 mg three times per day on Days X + 66 and X + 67, and then orally (CRESEMBA^®^ Capsules) once daily after breakfast (approximately 08:00), which is 2 h before cyclosporine administration. The blood cyclosporine level and the C/D ratio decreased after switching from voriconazole to isavuconazole; the blood cyclosporine level decreased from 124 ng/mL to a range of 58–86 ng/mL, and the C/D ratio decreased from 63 (ng/mL)/(mg/kg) to a range of 27–41 (ng/mL)/(mg/kg). After on Day X + 15, *Aspergillus* antigen was still negative.

**Figure 2:**
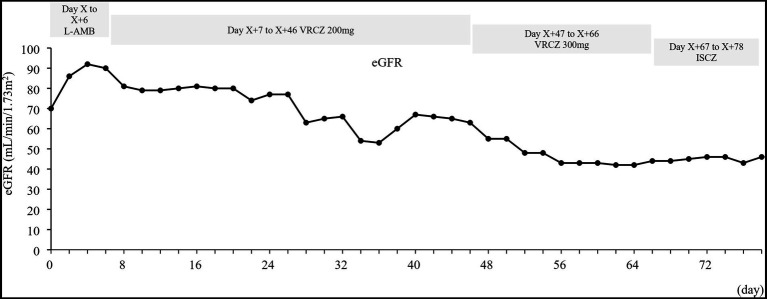
Time-related change in eGFR. eGFR, estimated glomerular filtration rate; ISCZ, isavuconazole; L-AMB, liposomal amphotericin B; VRCZ, voriconazole. The eGFR was calculated using the prediction equation for Japanese patients: eGFR (mL/min/1.73 m^2^) = 194 × SCr^−1.094^ × age^−0.287^ (×0.739 for females) (Matsuo et al., 2009).

The estimated glomerular filtration rate (eGFR) was calculated using the prediction equation for Japanese patients: eGFR (mL/min/1.73 m^2^) = 194 × SCr^−1.094^ × age^−0.287^ (×0.739 for females) (Matsuo et al., 2009). An increase in urine β 2-microglobulin, indicating renal tubular damage, was observed after administration of anti-human rabbit thymocyte immunoglobulin and liposomal amphotericin B (data not shown). In addition, a decrease in eGFR was noted during cyclosporine therapy from 70 mL/min/1.73 m^2^ on Day X to 46 mL/min/1.73 m^2^ on Day X + 78 (Figure 2). Hypomagnesemia (1.2–1.8 mg/dL) was also observed during the period of cyclosporine administration. Elevated blood cyclosporine levels were observed during period of voriconazole administration (Supplementary Table 1). Although the blood cyclosporine level decreased after switching from voriconazole to isavuconazole, monitoring was continued without increasing the cyclosporine dose due to potential effects on renal function.

The patient experienced vomiting (CTCAE Grade 2) on Day X + 79. Oral metoclopramide was prescribed and administered as required. Despite this treatment, the vomiting continued, resulting in a switch from isavuconazole to micafungin. Candida was also considered, and after the removal of the CV catheter, β-D-glucan levels was not improved. This case is considered one of intolerance to first-line therapy, and thus, micafungin monotherapy was initiated. β-D-glucan levels improved with micafungin monotherapy. However, nausea persisted, and magnetic resonance image of the head did not reveal any obvious intracranial lesions. On Day X + 82, the patient developed a fever of 40.9°C and had an elevated C-reactive protein level of 12.2 mg/dL, which led to suspicion of sepsis, and meropenem 1 g every 8 h was administered in addition to fluid replacement. Subsequently, *Klebsiella oxytoca* was detected in a blood culture. After de-escalation to cefepime on Day X + 90, antibiotic treatment was discontinued on Day X + 95. Following antibiotic treatment and fluid replacement, the vomiting ceased.

## 3 Materials and methods

### 3.1 Data collection

Total serum cyclosporine concentrations were determined using Dimension Xpand-HM with a Cyclosporine Flex reagent cartridge (Dade Behring Corporation, Tokyo, Japan) for antibody-conjugated magnetic immunoassay method. The quantitative range of cyclosporine for both instruments was 25.0–500.0 ng/mL.

### 3.2 PBPK model simulation

A PBPK model simulation was conducted using DDI simulator 2.6 (FUJITSU Limited, Kanagawa, Japan), incorporating *in vitro* and clinical data to assess the potential interaction between isavuconazole and cyclosporine. The constant inhibition *K*_i_ of half-maximal inhibitory concentration of isavuconazole on flumatinib in recombinant human CYP3A4 was 5.48 μM, which was referred from a previous report (Liu et al., 2023).

### 3.3 FAERS database study

Adverse event reports were downloaded from the FDA website (https://www.fda.gov/; accessed March 23, 2024). Duplicate reports were identified by matching case numbers, and the most recent reports were used as recommended by the FDA (Poluzzi et al., 2012). Data were extracted from the FAERS database covering the period between January 2004 and March 2024. Adverse events were described using terminology from the Medical Dictionary for Regulatory Activities (MedDRA, version 27.0), developed by the International Council on Harmonisation of Technical Requirements for Pharmaceuticals for Human Use. Adverse events were defined using MedDRA-conforming terminology. Drug-related adverse event reports were analyzed by excluding cases in which the role code was not classified as suspected primary or secondary. Reports with input errors such as a start date later than the event date were excluded. Adverse event reports related to drug-induced liver injury [Preferred Term (PT), 10072268], hyperlipidaemia (PT, 10062060), hypertension (PT, 10020772), renal failure (PT, 10038435), vomiting (PT, 10047700), nausea (PT, 10028813), diarrhoea (PT, 10012735), hyperglycaemia (PT, 10020635), hyperkalaemia (PT, 10020646), hyperuricaemia (PT, 10020903), tremor (PT, 10044565), gingival hypertrophy (PT, 10018284), visual impairment (PT, 10047571), hypertrichosis (PT, 10020864), thrombotic microangiopathy (PT, 10043645), and rash (PT, 10037844) (Kahan, 1989) were extracted. Reports of cyclosporine, cyclosporine concomitantly with isavuconazole, and cyclosporine concomitantly with voriconazole were collected. The collected data included the case number, drug name, adverse event name, start date of administration, end date of administration, and date of development of adverse event. The relationships between the combination of concomitant medications, cyclosporine and voriconazole or cyclosporine and isavuconazole, and adverse events were evaluated using the reporting odds ratio (ROR) with 95% confidence interval (CI) and univariate logistic regression analysis. The reference for the ROR was the cyclosporine only group. A statistically significant ROR was defined as a lower limit of the 95% CI exceeding 1.0. Statistical significance was set at *p* < 0.05.

## 4 Results

### 4.1 PBPK model simulation

The final verified models were used to predict changes in cyclosporine exposure following concomitant administration with isavuconazole or voriconazole. A PBPK model for isavuconazole was constructed by fitting a two-compartment model based on the pharmacokinetic plot diagram in the Japanese Interview Form of isavuconazonium sulfate (CRESEMBA^®^ Capsules), yielding the following parameters: renal clearance, 0.003 L/h; fraction absorbed (F_a_) intestinal availability (F_g_), 0.98; V_1_, 70.36; absorption rate constant, 1.51/h; hepatic clearance int., 254.19 L/h; K_12_, 0.30/h; and K_21_, 0.100/h. The residue plot in the PBPK model fit had a favorable Akaike information criterion of 151. The F_a_F_g_ in the registered cyclosporine PBPK model was 0.28. To assess the reliability of the PBPK model of isavuconazole, the serum concentration profiles obtained from a clinical study were compared with the concordance of the PBPK model constructed from the data. The results showed that the *C*_2_ values were 2,574 ng/mL and 2,652 ng/mL, respectively, and the AUC_0–12_ values were 16,221 ng·h/L and 16,118 ng·h/L, respectively, which were very well reproduced.

Considering the inhibitory effect of isavuconazole in the gastrointestinal tract, the predicted AUC and *C*_max_ values of cyclosporine (oral 50 mg × 2) with isavuconazole (oral 200 mg × 1) were 1.48-fold and 1.84-fold higher, respectively, than those without isavuconazole. When considering only the inhibition of hepatic metabolism and that F_a_F_g_ was unchanged by isavuconazole, the AUC and *C*_max_ were 1.009-fold and 1.010-fold higher, respectively, than those without isavuconazole.

The registered PBPK parameters were used for voriconazole. Considering the inhibitory effect in the gastrointestinal tract, the predicted AUC and *C*_max_ of cyclosporine (oral 50 mg × 2) concomitantly with voriconazole (30 mg × 2, predicted serum trough concentration approximately 1.0 μg/mL) were 3.74-fold and 3.86-fold higher, respectively than those without isavuconazole. With the inhibition of hepatic metabolism alone, the AUC and *C*_max_ were both 1.41-fold higher than those without isavuconazole.

### 4.2 FAERS database study

**Figure 3:**
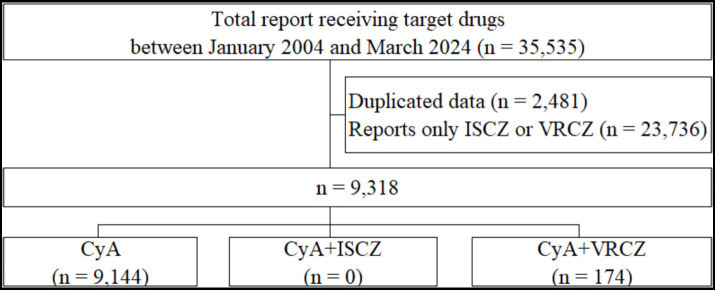
Flow chart of the process for data collection in the FAERS database study. CyA, cyclosporine; FAERS, Food and Drug Administration Adverse Event Spontaneous Reporting Database; ISCZ, isavuconazole; VRCZ, voriconazole.

In total, 35,535 reports were extracted from the FAERS database. The patient flowchart is shown in Figure 3. These reports included cyclosporine only (*n* = 9,144), a combination of cyclosporine and isavuconazole (*n* = 0), and a combination of cyclosporine and voriconazole (*n* = 174). Supplementary Table 2 shows the frequencies of adverse events reported for each group. The ROR for the combination of cyclosporine and isavuconazole could not be assessed because of insufficient reports. The RORs and 95% CIs for the variables in the cyclosporine with voriconazole group compared to the cyclosporine only group were as follows: drug-induced liver injury (ROR, 16.03; 95% CI, 4.37–58.74; *p* < 0.001), tremor (ROR, 2.82; 95% CI, 1.13–7.02; *p* = 0.026), and thrombotic microangiopathy (ROR, 4.57; 95% CI, 2.28–9.18; *p* = 0.003) (Supplementary Table 3).

## 5 Discussion

In our presented case, there was a decrease in the blood cyclosporine level by less than half after switching from voriconazole to isavuconazole, which is consistent with a previous report (Kozuch et al., 2024). This is attributed to a weaker interaction between isavuconazole and cyclosporine compared to that with voriconazole and cyclosporine. We investigated the adverse events associated with the combination of cyclosporine and isavuconazole using the FAERS database; however, because of a lack of reports, the ROR for cyclosporine and isavuconazole could not be determined.

The inserted packaging of isavuconazole in the US includes a cautionary warning regarding its use with cyclosporine, noting the potential for serious adverse events affecting the kidneys or brain due to an elevated blood cyclosporine level (European Medicines Agency, 2024). Neither US nor European Union labeling recommends empirical dose reductions of cyclosporine when initiating isavuconazole; however, both recommend monitoring and adjusting the blood cyclosporine level as required. In the present case, the blood cyclosporine level during isavuconazole administration was comparable to that during amphotericin B administration, which does not interact with CYP3A4. PBPK modeling indicated that hepatic inhibition alone results in a minimal increase in the blood cyclosporine level when cyclosporine and isavuconazole are concomitantly administered. Although intestinal and hepatic enzymes are major sites of interaction during oral administration, maintaining a 2 h interval between the administration of isavuconazole and cyclosporine may assist in reducing CYP3A4 drug interactions in the gastrointestinal tract. It is particularly important to consider drug–drug interactions when cyclosporine and isavuconazole are simultaneously orally administered.

In a Phase 3 clinical trial, fewer patients receiving isavuconazole experienced drug-related adverse events than those receiving voriconazole (42% vs. 60%) (Maertens et al., 2016). Regarding the medical cost, voriconazole is more expensive than isavuconazole (Harrington et al., 2017; Floros et al., 2019, 2020). In clinical trials, the common adverse events associated with isavuconazole include gastrointestinal issues such as nausea (26%) and vomiting (25%). In clinical trials, these symptoms are generally not severe enough to require the discontinuation of therapy (Maertens et al., 2016). Although our patient experienced vomiting during isavuconazole administration, this may have been caused by sepsis. The patient also experienced moderate renal failure during cyclosporine therapy. The patient had a history of smoking, hypertension, hypomagnesemia, and had previously received liposomal amphotericin B, all of which could contribute to renal failure (Caimi et al., 2014; Salvatore et al., 2015; Jaimes et al., 2007; Miura et al., 2002; Tashiro et al., 2022). Smoking is an independent predictor of microalbuminuria in patients with primary hypertension, potentially affecting renal failure through mechanisms, such as chronic endothelial dysfunction, oxidative stress, and glomerular hardening (Caimi et al., 2014; Salvatore et al., 2015). Nicotine inhalation has been shown to cause mesangial cell proliferation, which may contribute to renal failure (Jaimes et al., 2007). Cyclosporine-induced hypomagnesemia can exacerbate chronic renal fibrosis by upregulating fibrogenic molecules, notably through the early activation of tissue inhibitor of matrix metalloproteinase-1 expression (Miura et al., 2002). Chronic cyclosporine nephrotoxicity is often a result of excessive exposure, as indicated by higher AUC values (Paul and de Fijter, 2004). This patient also had an elevated blood cyclosporine level during voriconazole administration, which may have contributed to renal failure.

In the FAERS database analysis, there were no reports of adverse events in patients who were concomitantly administered isavuconazole and cyclosporine. In this case, renal failure was observed during concomitant administration of voriconazole and cyclosporine; however, the ROR was not significant in the FAERS data. There are significantly more reports of drug-induced liver injury, tremor, and thrombotic microangiopathy in patients receiving both cyclosporine and voriconazole than in those only receiving cyclosporine. Several case series of cyclosporine-related biliary sludge and cholelithiasis have been reported (Devarbhavi, 2012). At high doses, cyclosporine inhibits bile salt pumps and transporters, reduces bile flow, and potentially causes mild hyperbilirubinemia (Devarbhavi, 2012). Voriconazole-associated liver injury is a common condition that manifests as elevated liver enzyme levels that can lead to discontinuation of drugs (Chiang et al., 2024). Therefore, the increase in liver injury caused by the combination of cyclosporine and voriconazole may be attributed to voriconazole. In addition, previous studies have suggested that an elevated blood cyclosporine level (Berden et al., 1985) and concomitant administration of drugs that inhibit cyclosporine metabolism are risk factors for the development of tremor (Eidelman et al., 1991; Lucey et al., 1990) and thrombotic microangiopathy (Al-Nouri et al., 2015). As voriconazole is a strong CYP3A4 inhibitor, drug–drug interactions involving CYP3A4 may contribute to the increase in these adverse events.

This study had some limitations. First, as oral cyclosporine and isavuconazole were not administered simultaneously, it was not possible to quantify the extent of their interaction in the small intestine. Second, although renal function declined after the initiation of cyclosporine administration, it is unclear whether this decline was due to the blood cyclosporine level or the effects of anti-human rabbit thymocyte immunoglobulin and liposomal amphotericin B. Third, the blood cyclosporine level was not measured after the increase in voriconazole dosage; therefore, the impact of the blood voriconazole level on the blood cyclosporine level could not be assessed. Fourth, while the 2 h post-dose blood cyclosporine level correlated better than the trough level with the AUC (Cole et al., 2003), only the cyclosporine trough level was measured to guide dosing. Fifth, a PBPK model tailored to this patient has not been established. Sixth, in the FAERS database study, reports of adverse events were extracted that occurred during cyclosporine administration combined with either voriconazole or isavuconazole using the PTs derived from MedDRA terminology. Finally, the FAERS database lacks information on clinical laboratory data.

## 6 Conclusion

In the present case, the blood cyclosporine level decreased by less than half after switching from voriconazole to isavuconazole. This was attributed to a weaker interaction between isavuconazole and cyclosporine than between voriconazole and cyclosporine. However, it is advisable that there is a dosing interval of approximately 2 h between isavuconazole and cyclosporine to minimize the interaction in the intestine and cyclosporine-induced adverse events.

## Data Availability

The original contributions presented in the study are included in the article/Supplementary material, further inquiries can be directed to the corresponding author.
