# Supplementary material for: Changes in the blood cyclosporine level after switching from voriconazole to isavuconazole in a patient with aplastic anemia: insights from physiologically based pharmacokinetic model simulation and the Adverse Event Reporting System database study
Source: Front Microbiol. 2025 Feb 24;16:1525991. doi: 10.3389/fmicb.2025.1525991 (PMC11891238; doi:10.3389/fmicb.2025.1525991)
Supplement: Supplementary file 1 [file Table_1.docx]

Supplementary Table 1. Laboratory variables

| After cyclosporine administration (days) | | L-AMB | | | | | | | | |  | VRCZ | | | | | | | | | | | | | | | | | | | | | | | |  | ISCZ | | | | | | |
| --- | --- | --- | --- | --- | --- | --- | --- | --- | --- | --- | --- | --- | --- | --- | --- | --- | --- | --- | --- | --- | --- | --- | --- | --- | --- | --- | --- | --- | --- | --- | --- | --- | --- | --- | --- | --- | --- | --- | --- | --- | --- | --- | --- |
|  |  | 0 | 1 | 2 | 3 | 4 | 5 | 6 |  | 7 | | | 8 | 10 | 12 | 15 | 17 | 19 | 22 | 24 | 26 | 29 | 31 | 33 | 37 | 40 | 47 | 50 | 52 | 54 | 57 | 60 | 64 |  | 66 | | | 68 | 71 | 73 | 75 | 78 |  |
| CyA |  |  |  |  |  |  |  |  |  |  | | |  |  |  |  |  |  |  |  |  |  |  |  |  |  |  |  |  |  |  |  |  |  |  | | |  |  |  |  |  |  |
| Daily dose per body weight | mg/day | 60 | 60 | 60 | 100 | 100 | 100 | 100 |  | 100 | | | 100 | 100 | 100 | 70 | 70 | 70 | 90 | 120 | 90 | 90 | 100 | 100 | 100 | 100 | 120 | 100 | 100 | 100 | 100 | 90 | 90 |  | 90 | | | 90 | 100 | 100 | 100 | 100 |  |
| Blood level | ng/mL | 40 |  |  | 48 |  | 57 |  |  |  | | | 112 | 102 | 224 | 71 | 104 | 104 | 79 | 87 | 213 | 112 | 108 | 106 | 114 | 163 | 134 | 191 | 153 | 160 | 155 | 151 | 119 |  | 124 | | | 77 | 58 | 81 | 74 | 86 |  |
| C/D | (ng/mL)/(mg/kg) | 30 |  |  | 36 |  | 26 |  |  |  | | | 50 | 45 | 99 | 63 | 68 | 68 | 50 | 45 | 81 | 54 | 54 | 50 | 50 | 72 | 50 | 72 | 68 | 72 | 68 | 68 | 59 |  | 63 | | | 41 | 27 | 36 | 32 | 41 |  |
| Blood test |  |  |  |  |  |  |  |  |  |  | | |  |  |  |  |  |  |  |  |  |  |  |  |  |  |  |  |  |  |  |  |  |  |  | | |  |  |  |  |  |  |
| *β*-D-glucan | pg/mL |  |  |  | 13.4 |  |  |  |  |  | | |  | <6.0 |  |  | <6.0 |  |  | 8 |  |  | 9 |  |  |  |  |  | 15 |  |  | 23 | 44 |  |  | | |  | 25 |  |  | 24 |  |
| Alb | g/dL | 2.2 | 2.1 | 2.2 | 2.2 | 2.5 | 2.5 | 2.6 |  | 2.5 | | | 2.6 | 2.5 | 2.7 | 2.6 | 2.6 | 2.6 | 2.7 | 2.7 | 2.7 | 2.7 | 2.7 | 2.7 | 2.5 | 2.3 | 2.7 | 3.2 | 3.0 | 2.9 | 2.8 | 3.0 | 3.1 |  | 2.9 | | | 3.0 | 3.0 | 3.1 | 3.0 | 3.2 |  |
| BUN | mg/dL | 23.1 | 18.6 | 20.8 | 24.6 | 24.1 | 25.7 | 26.8 |  | 30 | | | 32 | 34 | 36 | 30 | 28 | 27 | 21 | 19 | 20 | 22 | 22 | 21 | 24 | 22 | 21 | 19 | 18 | 18 | 18 | 18 | 18 |  | 16 | | | 16 | 17 | 17 | 21 | 19 |  |
| Scr | mg/dL | 0.9 | 0.8 | 0.7 | 0.7 | 0.7 | 0.7 | 0.7 |  | 0.7 | | | 0.8 | 0.8 | 0.8 | 0.8 | 0.8 | 0.8 | 0.8 | 0.8 | 0.8 | 0.9 | 0.9 | 0.9 | 1.1 | 0.9 | 1.0 | 1.1 | 1.2 | 1.2 | 1.3 | 1.3 | 1.4 |  | 1.3 | | | 1.3 | 1.3 | 1.3 | 1.3 | 1.3 |  |
| eGFR | mL/min/1.73m^2^ | 70 | 79 | 86 | 89 | 92 | 90 | 90 |  | 89 | | | 81 | 79 | 79 | 81 | 81 | 80 | 74 | 77 | 77 | 63 | 65 | 66 | 53 | 67 | 63 | 55 | 48 | 48 | 43 | 43 | 42 |  | 44 | | | 44 | 45 | 46 | 43 | 46 |  |
| K | mmol/L | 3.8 | 3.7 | 3.9 | 3.8 | 3.3 | 2.9 | 3.5 |  | 3 | | | 4 | 4 | 4 | 4 | 4 | 4 | 3 | 3 | 3 | 3 | 3 | 3 | 3 | 3 | 4 | 4 | 4 | 3 | 3 | 3 | 4 |  | 3 | | | 3 | 3 | 4 | 4 | 4 |  |
| AST | U/L | 33 | 15 | 17 | 21 | 104 | 64 | 48 |  | 61 | | | 77 | 61 | 50 | 15 | 16 | 10 | 10 | 13 | 14 | 20 | 20 | 19 | 7 | 21 | 13 | 11 | 9 | 9 | 8 | 9 | 9 |  | 9 | | | 9 | 9 | 13 | 12 | 9 |  |
| ALT | U/L | 76 | 54 | 50 | 56 | 177 | 214 | 196 |  | 209 | | | 250 | 242 | 215 | 107 | 78 | 58 | 42 | 38 | 37 | 42 | 47 | 46 | 21 | 33 | 30 | 19 | 11 | 10 | 6 | 7 | 5 |  | 6 | | | 7 | 11 | 17 | 16 | 11 |  |
| γ－GTP | U/L | 220 | 174 | 156 | 134 | 185 | 180 | 166 |  | 171 | | | 192 | 198 | 212 | 176 | 159 | 149 | 124 | 118 | 106 | 100 | 92 | 89 | 75 | 68 | 59 | 61 | 51 | 48 | 44 | 45 | 41 |  | 40 | | | 43 | 45 | 45 | 42 | 43 |  |
| T-Bil | mg/dL | 1.2 | 1.3 | 0.8 | 0.8 | 1.3 | 1.2 | 1.0 |  | 1.0 | | | 1.3 | 1.2 | 1.6 | 1.4 | 1.5 | 1.4 | 1.3 | 1.0 | 1.0 | 1.0 | 0.9 | 1.1 | 0.9 | 1.0 | 1.1 | 1.2 | 0.9 | 0.9 | 0.9 | 0.9 | 0.8 |  | 1.0 | | | 1.0 | 1.0 | 1.0 | 0.7 | 1.0 |  |
| Mg | mg/dL | 1.6 | 1.6 | 1.6 | 2.3 | 1.8 | 1.4 | 1.4 |  | 1.8 | | | 1.5 | 1.4 | 1.4 | 1.4 | 1.4 | 1.4 | 1.7 | 1.3 | 1.8 | 1.3 | 1.3 | 1.4 | 1.5 | 1.2 | 1.9 | 1.3 | 1.8 | 1.3 | 1.3 | 1.2 | 1.7 |  | 1.2 | | | 1.5 | 1.5 | 1.5 | 1.5 | 1.6 |  |
| Urinary *β* 2-microglobulin | μg/L |  |  | 1.5 |  |  |  |  |  |  | | |  | 1.7 |  |  | 1.5 |  |  | 0.9 |  |  | 0.7 |  | 0.7 |  |  |  | 0.7 |  |  | 0.9 |  |  |  | | |  | 1.6 |  |  |  |  |
| Urine protein-to-creatinine ratio | g/g・Crea |  |  | 67061 |  |  |  |  |  |  | | |  | 36025 |  |  | 23387 |  |  | 26511 |  |  | 11973 |  | 39606 |  |  |  | 36969 |  |  | 18395 |  |  |  | | |  | 42550 |  |  |  |  |
| Concomitant medication |  |  |  |  |  |  |  |  |  |  | | |  |  |  |  |  |  |  |  |  |  |  |  |  |  |  |  |  |  |  |  |  |  |  | | |  |  |  |  |  |  |
| Daily L-AMB dose | mg/day | 200 | 200 | 200 | 200 | 200 | 200 | 200 |  |  | | |  |  |  |  |  |  |  |  |  |  |  |  |  |  |  |  |  |  |  |  |  |  |  | | |  |  |  |  |  |  |
| Daily VRCZ dose | mg/day |  |  |  |  |  |  |  |  | 200 | | | 200 | 200 | 200 | 200 | 200 | 200 | 200 | 200 | 200 | 200 | 200 | 200 | 200 | 200 | 300 | 300 | 300 | 300 | 300 | 300 | 300 |  |  | | |  |  |  |  |  |  |
| Blood VRCZ level | μg/mL |  |  |  |  |  |  |  |  |  | | |  |  | 1.0 |  |  |  |  |  |  |  |  |  |  | 0.6 |  |  |  |  |  |  |  |  |  | | |  |  |  |  |  |  |
| Daily ISCZ dose | mg/day |  |  |  |  |  |  |  |  |  | | |  |  |  |  |  |  |  |  |  |  |  |  |  |  |  |  |  |  |  |  |  |  | 600 | | | 200 | 200 | 200 | 200 | 200 |  |
| Daily rATG dose | mg/day |  | 112.5 | 112.5 | 112.5 | 112.5 | 112.5 |  |  |  | | |  |  |  |  |  |  |  |  |  |  |  |  |  |  |  |  |  |  |  |  |  |  |  | | |  |  |  |  |  |  |

Alb, serum albumin; ALT, alanine aminotransferase; AST, aspartate aminotransferase; BUN, blood urea nitrogen; C/D, cyclosporine concentration/dose-normalized body weight; CyA, cyclosporine: eGFR, estimated glomerular filtration rate; ISCZ, isavuconazole; L-AMB, Liposomal amphotericin B; γ－GTP, γ-glutamyl transpeptidase; K, potassium; Scr, serum creatinine; Mg, magnesium, rATG, rabbit antithymocyte globulin; T–Bil, total bilirubin; VRCZ, voriconazole

The C/D ratio was calculated as follows: $C/D (ng/mL)/(mg/kg)=(blood cyclosporine level)/(daily \mathrm{cyclosporine}dose per body weight)$.

eGFR was calculated using the prediction equation for Japanese patients: eGFR (mL/min/1.73 m^2^) = 194 × SCr^−1.094^ × age^−0.287^ (×0.739 if female) (Matsuo et al., 2009).
