# Supplementary material for: Changes in the blood cyclosporine level after switching from voriconazole to isavuconazole in a patient with aplastic anemia: insights from physiologically based pharmacokinetic model simulation and the Adverse Event Reporting System database study
Source: Front Microbiol. 2025 Feb 24;16:1525991. doi: 10.3389/fmicb.2025.1525991 (PMC11891238; doi:10.3389/fmicb.2025.1525991)
Supplement: Supplementary file 2 [file Table_2.docx]

Supplementary Table 2. Adverse event report (FAERS)

|  | CyA | CyA + ISCZ | CyA + VRCZ |
| --- | --- | --- | --- |
|  | n = 9,144 | n = 0 | n = 174 |
| Drug-induced liver injury | 10 (0.1) | 0 (NaN) | 3 (1.7) |
| Hyperlipidemia | 34 (0.4) | 0 (NaN) | 1 (0.6) |
| Hypertension | 284 (3.1) | 0 (NaN) | 6 (3.4) |
| Renal failure | 446 (4.9) | 0 (NaN) | 8 (4.6) |
| Vomiting | 289 (3.2) | 0 (NaN) | 3 (1.7) |
| Nausea | 277 (3.0) | 0 (NaN) | 6 (3.4) |
| Diarrhea | 376 (4.1) | 0 (NaN) | 2 (1.1) |
| Hyperglycemia | 42 (0.5) | 0 (NaN) | 2 (1.1) |
| Hyperkaliemia | 78 (0.9) | 0 (NaN) | 1 (0.6) |
| Hyperuricemia | 31 (0.3) | 0 (NaN) | 2 (1.1) |
| Tremor | 95 (1.0) | 0 (NaN) | 5 (2.9) |
| Gingival hypertrophy | 14 (0.2) | 0 (NaN) | 0 (0.0) |
| Visual impairment | 27 (0.3) | 0 (NaN) | 0 (0.0) |
| Hypertrichosis | 14 (0.2) | 0 (NaN) | 0 (0.0) |
| Thrombotic microangiopathy | 108 (1.2) | 0 (NaN) | 9 (5.2) |
| Rash | 161 (1.8) | 0 (NaN) | 3 (1.7) |

CyA, cyclosporine: FAERS, the Adverse Event Spontaneous Reporting Database; ISCZ, isavuconazole;

NaN, not a number; VRCZ, voriconazole

CI, confidence interval; CyA, cyclosporine: FAERS, the Adverse Event Spontaneous Reporting Database;

ISCZ, isavuconazole; ROR, reporting odds ratio; VRCZ, voriconazole
