# Supplementary material for: Changes in the blood cyclosporine level after switching from voriconazole to isavuconazole in a patient with aplastic anemia: insights from physiologically based pharmacokinetic model simulation and the Adverse Event Reporting System database study
Source: Front Microbiol. 2025 Feb 24;16:1525991. doi: 10.3389/fmicb.2025.1525991 (PMC11891238; doi:10.3389/fmicb.2025.1525991)
Supplement: Supplementary file 3 [file Table_3.docx]

Supplementary Table 3. Risk analyses of adverse events of reports (FAERS)

|  | CyA + ISCZ | | | CyA + VRCZ | |
| --- | --- | --- | --- | --- | --- |
|  | ROR [95% CI] | *p*–value | ROR [95% CI] | | *p*–value |
| Drug-induced liver injury | – | – | 16.03 [4.37–58.74] | | <0.001 |
| Hyperlipidemia | – | – | 1.55 [0.21–11.38] | | 0.667 |
| Hypertension | – | – | 1.11 [0.49–2.54] | | 0.797 |
| Renal failure | – | – | 0.94 [0.46–1.92] | | 0.865 |
| Vomiting | – | – | 0.54 [0.17–1.69] | | 0.289 |
| Nausea | – | – | 1.14 [0.50–2.60] | | 0.750 |
| Diarrhea | – | – | 0.27 [0.07–1.10] | | 0.067 |
| Hyperglycemia | – | – | 2.52 [0.61–10.49] | | 0.204 |
| Hyperkaliemia | – | – | 0.67 [0.09–4.86] | | 0.694 |
| Hyperuricemia | – | – | 3.42 [0.81–14.40] | | 0.094 |
| Tremor | – | – | 2.82 [1.13–7.02] | | 0.026 |
| Gingival hypertrophy | – | – | 1.80 [0.11–30.37] | | 0.682 |
| Visual impairment | – | – | 0.95 [0.06–15.64] | | 0.971 |
| Hypertrichosis | – | – | 1.80 [0.11–30.37] | | 0.682 |
| Thrombotic microangiopathy | – | – | 4.57 [2.28–9.18] | | 0.003 |
| Rash | – | – | 0.98 [0.31–3.10] | | 0.971 |

CI, confidence interval; CyA, cyclosporine: FAERS, the Adverse Event Spontaneous Reporting Database;

ISCZ, isavuconazole; ROR, reporting odds ratio; VRCZ, voriconazole
